# Motivating Protective Behavior against COVID-19: Fear Versus Hope

**DOI:** 10.1177/08982643221089427

**Published:** 2022-06-17

**Authors:** Gregor Sand, Johanna Bristle

**Affiliations:** 1 Max Planck Institute for Social Law and Social Policy (MPISOC), Munich Center for the Economics of Aging (MEA), Munich, Germany

**Keywords:** protective behavior, COVID-19, protection motivation theory, fear and hope, SHARE

## Abstract

**Objectives:**

Based on protection motivation theory, we investigate how indicators of threat perception (perceived severity, perceived vulnerability, and fear arousal) and coping appraisal (hope) are associated with older people’s motivation to engage in protective behavior after the outbreak of COVID-19.

**Methods:**

We use multivariate regression analyses with a sample of 40,282 individuals from 26 countries participating in the SHARE Corona Survey.

**Results:**

We find that 15% of all respondents stayed home completely—mainly the oldest and vulnerable people with prior health risk conditions. On average, older Europeans responded strongly to the recommended protective behavior measures (6 out of 7 measures adopted). Among the threat perception indicators, fear arousal is the main motivator for protective behavior, whereas the coping appraisal indicator hope shows an equally strong association.

**Discussion:**

Given the negative health effects of fear, our findings may help evaluate and revise governmental policy responses and communication strategies.

## Introduction

Throughout previous pandemics, prevention measures had been largely the same. For example, as early as the Spanish flu of 1918, there were non-pharmaceutical interventions (NPIs) such as school closures, restrictions of public gatherings, quarantine, health education, and personal hygiene from hand washing to wearing face masks (Balinska & Rizzo, 2009). After the outbreak of COVID-19, governmental policy responses to control the virus and protect the most vulnerable groups—older adults and those with underlying health conditions—have put a strong emphasis on national lockdowns, social distancing, and other NPIs (Jordan et al., 2020). These control measures have polarized people and affected many facets of people’s lives and societies in terms of non-intended health, economic, and social consequences. Most of these consequences will become visible over time. This study already provides insight into people’s immediate response to the threat based on their perceptions, feelings, and behavioral changes.

### Extant Research

Previous research on threat perceptions has shown that people tend to have a distorted perception of the severity of threats, especially in scenarios with high uncertainty. For instance, analyses by Slovic et al. (1980) on the individual risk assessment of the seriousness of threats revealed that people usually overestimate the number of deaths due to natural disasters such as earthquakes, but they underestimate the number of deaths due to common diseases like diabetes or asthma. Extant studies on risk perceptions during pandemics have found that people who perceive themselves to be more vulnerable or susceptible to the threat engage more in protective behavior (e.g., study by Brug et al. (2004) on people’s risk perceptions, knowledge, and precautions during SARS-CoV-1 in 2003). With respect to the individual assessment of fear, Jones and Salathé (2009) demonstrated that anxiety increased the likelihood to engage in protective behavior in response to swine flu in 2009. Recent studies by Harper et al. (2020), Jørgensen et al. (2021), and Yıldırım et al. (2021) have shown similar results for COVID-19. Jørgensen et al. (2021) emphasize that even though fear works to predict compliance with recommended protective behavior, there are negative implications of anxiety for mental health and society. Other researchers have also illustrated the disruptive impact of fear and stress on people’s physical and mental health—reaching from immune system dysfunction to suicidal thoughts (Ahorsu et al., 2020; Brod, Rattazzi, Piras, & D'Acquisto, 2014; Nechita et al., 2018; Seiler et al., 2020; Şimşir et al., 2021). According to Kompaniyets et al. (2021), along with obesity and diabetes, anxiety and fear-related disorders are the strongest risk factors for severe COVID-19 illness and death. While there is little evidence that individuals with higher interpersonal and institutional trust are more likely to engage in protective behavior, Jørgensen et al. (2021) maintain that knowledge-based efficacy (i.e., knowing about the virus and how to protect oneself well) provides “a pathway to compliance without fear” (p. 18). In this direction, other studies have detected that self-efficacy (i.e., the belief in one’s own ability to succeed) was associated with anxiety and self-protective behavior against COVID-19 (e.g., Graf et al., 2021). But self-efficacy is also associated with hope. In contrast to fear, hope has the potential to sustain commitment and coping (e.g., Lazarus, 1999; Nabi & Gall Myrick, 2019; Smith & Lazarus, 1990). Charles (2010) argues that better and more flexible coping strategies are related with older age—mainly due to emotion regulation strategies resulting from the experience, knowledge, and positivity gained in dealing with the problems of daily life. Only fear can disrupt this age-related propensity. Apart from people’s risk perceptions and emotional states, sociodemographic factors such as age, sex, education, occupation, region, migration background, and social contacts have been among the determining factors for protective behavior during the swine flu and COVID-19 (Jones & Salathé, 2009; Qiu et al., 2020).

### Research Question

In this study, we use data from the first Corona Survey of the SHARE project (Survey of Health, Ageing and Retirement) to explore how older European citizens differ in terms of adopting nationally recommended behavior to protect against COVID-19. The survey was fielded simultaneously in 26 European countries plus Israel, primarily in June and July 2020. Therefore, we measure respondents’ protective behavior patterns not during the first national lockdowns in spring 2020, but 2 to 3 months later. Even though the virus has affected all people, the motivation for protective behavior may be different among different age groups. While the main motivating factor among younger people is a pro-social orientation (Franzen & Woehner, 2021), the motivation to self-protect increases with age because most older people perceive themselves as more vulnerable and at risk (Brug et al., 2004; Jørgensen et al., 2021; Yıldırım et al., 2021). Distinguishing between older age groups can be useful since other studies (e.g., Litwin & Levinsky, 2021; Pasion et al., 2020) even found that the oldest age group (age 70 or more) reported lower perceived risk and showed less protective behavior compared with younger old people (age 60–69).

The primary research interest is in the relationship of individuals’ threat perceptions (with regard to the severity of the virus, one’s vulnerability, and feelings of fear) and coping appraisal (i.e., capability to deal with the threat captured by expressing hope through optimistic statements). By including data on governmental control measures and mortality rates, we examine whether macrolevel factors help explain potential country disparities. To our knowledge, none of the existing studies on threat perceptions, coping appraisal, and protection during the COVID-19 crisis have used a theory-driven approach that combines self-reports and aggregate-level indicators in a cross-country perspective.

### Theory

We borrow aspects from two types of concepts of risk perception: the psychometric paradigm and expectancy–value models. While the former focuses on mapping people’s perceptions of different types of risks and hazards via assessment instruments, the latter explore how individuals’ risk perceptions affect their behavior. One of the key aspects in this study is the role of fear with regard to individual threat appraisals. Within the psychometric paradigm, Roseman et al. (1996) hold that fear is elicited by events that are appraised as motive-inconsistent, unknown, unexpected, and uncertain. According to Slovic et al. (1980), dimensions such as familiarity (characterized by attributes such as observability, knowledge, and immediacy of consequences), dread (characterized by attributes such as uncontrollable severity, catastrophic, involuntary, and threat to human future), and exposure (i.e., number of people exposed) play an important role in people’s risk perception. Leppin and Aro (2009) attribute a high unknown risk and dread risk to hazards such as pandemics since they are hard to observe and control, catastrophic, fairly unknown to science, and their effects are delayed. Therefore, we assume that COVID-19 poses an unknown threat that has stimulated negative emotions and uncertainty at a global scale, especially at its inception.

Protection motivation theory (PMT) is a social cognition theory developed to understand the impact of fear appeals on behavioral change, that is, how people respond to health threats (Conner & Norman, 2005; Leppin & Aro, 2009; Ling et al., 2019; Milne et al., 2000). Fear appeals refer to informative communication about a threat with regard to how threatened one feels and how to deal with the threat. While human behavior is shaped and influenced by intrapersonal characteristics and environmental or contextual conditions, the likelihood to engage in protective behavior is also determined by the beliefs, appraisals, or perceptions that people have about the threat itself and about engaging in the desired protective behavior. Individuals who appraise potential stressors as more threatening (e.g., those who are personally at risk) are more motivated to protect themselves (Conner & Norman, 2005; Floyd et al., 2000; Ling, et al., 2019; Milne et al., 2000; Rogers, 1975, 1983). Intention is the best predictor of protective behavior and determined by two parallel cognitive and partly emotional processes: threat appraisal and coping appraisal (Conner & Norman, 2005; Ling et al., 2019).

Threat appraisal captures individuals’ evaluation of the components of a fear appeal related to how threatened one feels. All of them are expected to increase protective behavior: perceived severity (i.e., individual assessment on the seriousness of the threat to oneself), perceived vulnerability (i.e., individual assessment on the susceptibility of oneself to the threat), and fear arousal (i.e., individual assessment of the fear the threat evokes for oneself) (Floyd et al., 2000; Milne et al., 2000; Slovic et al., 1980). In this study, we refer to fear as a self-reported, situational, affective, or emotional state (i.e., state anxiety). Hence, it refers to a present-moment assessment of physiological and emotional symptoms associated with anxiety and not a personal trait that describes the predisposition to react with anxiety in stressful situations (i.e., trait anxiety) (see Wirtz et al., 2019). “The greater the perceived threat, the more likely the individual is to be motivated to protect himself or herself …” (Milne et al., 2000, p. 109). In the case of COVID-19, we assume that fear arousal as the affective or emotional component of threat appraisal belongs to the main determinants for people’s risk perceptions and motivation to engage in protective behavior. However, neither theory nor data allow us to differentiate between the underlying causes of fear arousal. Perceiving the virus as a concrete health threat may motivate older and/or vulnerable people to protect themselves, whereas considering the virus and all related control measures as a financial or sociocultural threat might trigger adverse behavior (see Kachanoff et al., 2020). In this study, we can only account for the association of COVID-19 as a health threat and individuals’ behavioral response.

Coping appraisal refers to individuals’ evaluation of the component of a fear appeal related to how one can deal or cope with a threat. It usually captures individuals’ beliefs about their response efficacy and self-efficacy, that is, whether they consider the recommended protective behavior effective in reducing the threat and whether they consider themselves able to perform the recommended protective behavior. In short, response and self-efficacy describe individuals’ perceived capability to cope with a threat. The involved response costs (e.g., expenses, penalties, time, and effort) can decrease the adoption of protective behavior (Floyd et al., 2000; Milne et al., 2000). According to Ling et al. (2019), people engage most in protective behavior when they believe that non-engagement poses a threat to themselves (high threat appraisal) and when they believe that their behavior can reduce the threat (high coping appraisal). In the light of COVID-19, the former may apply especially to individuals that are personally affected and/or belong to the high-risk group, the latter to individuals who confide in the effectiveness of governmental and/or their own coping strategies. In this analysis, we are forced to deviate from the theoretical construct because the dataset lacks established indicators for coping appraisal. Instead, we use hope as a proxy measure for coping appraisal. We argue that expressing hope through optimistic statements regarding the situation and expectations about the future is closely related to response and self-efficacy. Hope therefore represents the best available approximation for individual coping appraisal in the present data. This reasoning is substantiated by several studies mentioned above (Lazarus, 1999; Nabi & Myrick, 2019; Smith & Lazarus, 1990). They find a positive correlation between hope and self-efficacy and therefore argue that unlike fear, hope can be characterized by its coping potential. Since we do not intend to test the construct validity—which has been done by many other studies (e.g., Chen et al., 2009; Xiao et al., 2014)—we believe that we can draw on the determining factors of PMT as the theoretical foundation of our research.

**Figure 1.**
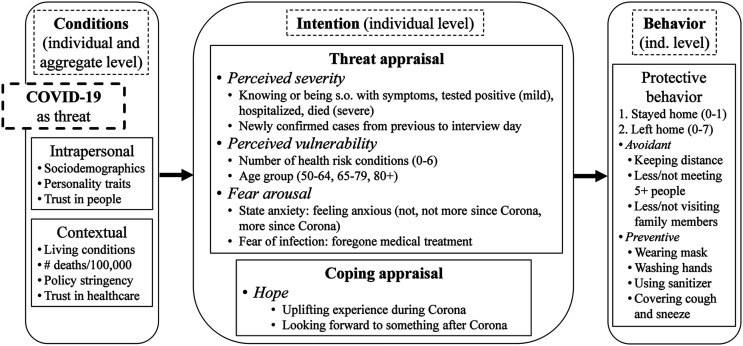
Illustration of theoretical framework including measures. Source: Own illustration.

Based on Figure 1 (see the “Measures” section below) and our theoretical underpinnings, we expect that respondents’ intention to engage in protective behavior depends on the threat itself as well as intrapersonal and contextual conditions and increases with individuals’:

### - Threat Appraisal

o H1: perceived severity of COVID-19 (severity hypothesis)
o H2: perceived vulnerability to the threat (vulnerability hypothesis)
o H3: fear arousal (fear hypothesis)

### - Coping Appraisal

o H4: hope expressed through optimistic statements (hope hypothesis)

## Methods

### Data and Sample

The present study is conducted with the SHARE Wave 8 COVID-19 data (Release 1.0.0, Börsch-Supan, 2021a; 2021b) from the first SHARE Corona Survey, augmented by variables from all regular SHARE waves from Release 7.1.0 (Börsch-Supan, 2020a; 2020b; 2020c; 2020d; 2020e; 2020f) and the Wave 8 Release 1.0.0 (Börsch-Supan, 2021c). The SHARE Corona Survey was conducted via computer-assisted telephone interviews (CATI) in 27 countries: Belgium, Bulgaria, Croatia, Cyprus, the Czech Republic, Denmark, Estonia, Finland, France, Greece, Germany, Hungary, Italy, Latvia, Lithuania, Luxembourg, Malta, the Netherlands, Poland, Portugal, Romania, Slovakia, Slovenia, Spain, Sweden, Switzerland, and Israel (Scherpenzeel et al., 2020). In contrast to many other studies, fieldwork was carried out from June to August 2020, months that were characterized by a slight relaxation of restrictions in most countries after the (partly) severe containment measures in spring 2020. Overall, 98% of all interviews were conducted in June or July 2020. In addition to the SHARE data, we include macrolevel information on the spread of the virus and governmental containment strategies from the Oxford COVID-19 Government Response Tracker (Hale et al., 2021).

We employ multiple logistic and linear regression analysis with robust standard errors. Our study population are European citizens aged 50 and older living in private households. Our analytical sample excludes respondents from the Netherlands due to a different survey mode in prior waves. After deleting cases with missing information on all variables of interest, our analysis sample consists of 40,282 persons from 25 European countries plus Israel. All analyses are conducted using Stata version 14.2.

### Measures

Figure 1 shows how our measures are embedded in the theoretical framework. In our analyses, we investigate how the components of threat appraisal and coping appraisal (middle panel) influence protective behavior (right panel). The conditions mentioned in the left panel serve as anticipatory controls or interpretive guidance (intrapersonal and contextual variables).

The subjectivity of measures plays a crucial role in explaining social behavior during uncertain events such as the COVID-19 crisis. In line with other studies (e.g., Ahorsu et al., 2020; Harper et al., 2020), people in fear may not be able to think rationally, and (negative) emotions could have a great influence on their actions. Therefore, we build on respondents’ appraisals using indicators for all of the subjective components of the PMT construct, at the same time controlling for several objective individual and contextual measures that are mentioned in the theory.

For our first outcome variable, we distinguish between respondents who stayed at home (“Stayed home”) and those who left their home (“Left home”) since the outbreak of COVID-19 until the day of interview (question wording: “Since the outbreak of Corona, have you ever left your home?”). Since stay-at-home orders were proclaimed as a protective measure in most countries and in some even mandatory (Ritchie et al., 2020c), we use this as binary outcome variable and distinguish between additional forms of protective behavior. Therefore, we take the subsample of respondents who left their home and create an additive index for protective behavior as the second outcome variable. It accounts for seven protective measures generally recommended by official representatives: avoidant behavior such as keeping distance (“Often” or “Always”); contact reduction measures such as visiting other family members and meeting with more than five people from outside of the household (“Less often” or “Not any more”); preventive behavior such as wearing a face mask (“Often” or “Always”); and hygiene measures such as washing hands, using special hand sanitizer or disinfection fluids, and covering one’s mouth when coughing or sneezing more frequently than usual (“Yes”). For all answers listed in parenthesis, we assign one point per respondent to the index. Hence, the second outcome variable “Protective Behavior Index” is an index score which can range between 0 and 7 and comprises the subsample of those who left home.

Our explanatory variables measure respondents’ threat perceptions and coping appraisal. With regard to threat perceptions, we distinguish between three indicators: perceived severity, perceived vulnerability, and fear arousal. Two indicators capture perceived severity. While the number of newly confirmed cases^1^ from the previous to the interview day enters the model log-transformed as an indicator for increased geographical closeness and potential exposure to the virus, direct exposure is measured by whether a respondent reports to be (i.e., self-exposure) or to know someone (i.e., network exposure) with symptoms, tested positive, hospitalized, or reports to know someone who died with COVID-19 (question wording example: “Have you or anyone close to you been hospitalized due to an infection from the Corona virus?”). The first two indicators are categorized as “mild” and the two last ones as “severe.” Multiple answers are assigned according to the most severe response category. Since Litwin and Levinsky (2021) show that self-exposure and network-exposure are similarly associated with protective behavior in terms of direction, significance, and magnitude, we do not distinguish between these two types of exposure.

Perceived vulnerability is measured by the number of health risk conditions (0–6) obtained from respondents’ last available regular SHARE wave data. The risk conditions include being in need for home care and having health conditions such as hypertension, diabetes, cardiovascular disease, chronic respiratory disease, or a weakened immune system. The variable enters the models categorized. It is truncated to 3 due to low case numbers in the upper categories. In addition, age is added as vulnerability measure with the age categories 50–64 (occupationally active), 65–79 (young retirees), and 80+ (oldest old). In order to account for the connection of respondents’ vulnerability in terms of health risks and age, we introduce an interaction term of the categorical health risk variable and the age categories.

We use two indicators for fear arousal. State anxiety is measured with a one-item question asking “In the last month, have you felt nervous, anxious, or on edge?” Note that the time of reference ranges from the beginning of May to mid-July 2020, depending on the day of interview. It is the first item of the Generalized Anxiety Disorder Questionnaire (GAD-7) used as a screening tool and severity measure for generalized anxiety disorder. Since this question is kept general, we also use the COVID-19–specific follow-up question: “Has that been more so, less so, or about the same as before the outbreak of Corona?” We take both items and generate a categorical variable with the following three categories: “Not anxious,” “Anxious, but not more than before Corona,” and “Anxious, more than before Corona” (the latter based on answer “More so”). The second indicator is fear of infection, a binary variable that captures if a medical treatment was forgone due to COVID-19 (question wording: “Since the outbreak of Corona, did you forgo medical treatment because you were afraid to become infected by the Corona virus?”).

Hope as proxy measure for coping appraisal is operationalized by two binary indicators describing whether respondents expressed their hope through optimistic statements by naming an uplifting and hope-inspiring experience since the outbreak of COVID-19 (“What was your most uplifting experience since the outbreak of Corona, in other words, something that inspired hope or happiness?”) and by naming something to look forward to once COVID-19 abates (“…, what is it that you are looking most forward to doing once Corona abates?”). While both question items are open-ended, only the answer pattern is recorded and entered into our models as binary variables. Once a respondent named something quickly or after a while according to the assessment of the interviewer, we coded the corresponding variable with the value 1 (as opposed to 0, which reflects that a respondent did not name anything). We prefer the label hope to optimism because both items ask for hopeful experiences or events.

Based on PMT, we consider the following control variables as relevant influential factors in this analysis. We measure sociodemographic background and intrapersonal characteristics by including information on sex, marital status, migration background, education, home ownership, financial hardship during COVID-19, and employment status. Financial hardship during COVID-19 is measured as a variable with three categories that identify whether respondents did not report any financial difficulties, experienced financial difficulties, or postponed regular payments; dipped into savings; and/or lost their job or closed their business. In addition, we draw on respondents’ personality traits: openness, conscientiousness, extraversion, agreeableness, and neuroticism taken from the Big Five Inventory (Rammstedt & John, 2007), and general trust in people (on a scale from 0 to 10). As living conditions, we add respondents’ household size and information on whether they live in an urban or rural area. Regardless of the stay-home regulations, respondents’ mobility may have been restricted already prior to COVID-19, especially among the oldest age group. To account for such an alternative cause, we added a control variable that captures limitations prior to COVID-19 such as leaving the house independently, accessing public transportation services, and shopping for groceries. As we use the most recent data available for each respondent, we account for a time lag of the control variables by including an indicator if respondent info is drawn from Wave 8 (directly before the outbreak of COVID-19) or from previous waves. Finally, we add country dummies to account for country-specific effects.

In order to evaluate country differences based on national conditions at the macrolevel, we rely on an indicator for COVID-19 mortality expressed by the number of confirmed deaths per 100,000 inhabitants and a measure for governments’ policy stringency on a scale from 0 to 100. Both are taken from the Oxford COVID-19 Government Response Tracker (Hale et al., 2021; Ritchie et al., 2020a, 2020b). The data on mortality and stringency was taken from the date the interview took place. In addition, we draw on the idea of institutional trust by Jørgensen et al. (2021) and include a domain-specific trust indicator for institutional trust in the healthcare system based on Special Eurobarometer 411, Wave 80.2, fielded in 2013 (European Commission, 2014). We argue that it is important to examine to what extent individuals believe that they are treated well in case of having severe COVID-19 symptoms.

## Results

### Sample Description

In Table 1, we present all sample characteristics by reporting percentages or means with standard deviations (SD) for all variables that enter the regression models (except country dummies) for both analytical samples. Country characteristics are presented separately in Table 2. All indicators are weighted. The overall sample consists of 40,282 individuals aged 50+ in 26 countries. It can be seen that 6,976 respondents (15.7%) stayed at home completely between the outbreak of COVID-19 and the day of interview. The “Protective Behavior Index left-home subsample” comprises 33,306 individuals. Respondents from this subsample adopted on average six from seven recommended protection measures. The samples differ significantly in several characteristics (indicated in the last column of Table 1 based on a t-test, p-value levels displayed).

**Table 1.** Descriptive Statistics by Sample Type (presented as percentages or means with standard deviations).

	Overall sample		Stayed-home subsample		Left-home subsample		
Characteristics	%/Mean (SD)		%/Mean (SD)		%/Mean (SD)		t-test
*Outcomes*							
Stayed home (ref: left home)	15.66						
Protective Behavior Index					5.95 (1.21)		
*Threat appraisal: perceived severity*							
Not exposed	83.47		90.54		82.16		***
Mildly exposed (symptoms/tested positive)	11.54		6.21		12.53		***
Severely exposed (hospitalized/died)	4.99		3.24		5.31		***
Change in confirmed cases	185.09 (314.22)		150.00 (290.69)		191.37 (317.97)		***
*Threat appraisal: perceived vulnerability*							
No health risk conditions	54.18		42.01		56.44		***
1 health risk condition	29.83		31.81		29.47		**
2 health risk conditions	12.25		18.74		11.04		***
3+ health risk conditions	3.73		7.44		3.04		***
Age < 65	43.37		21.68		47.39		***
Age 65–79	41.06		42.14		40.86		
Age 80+	15.58		36.18		11.75		***
*Threat appraisal: fear arousal*							
Not feeling anxious	70.25		63.09		71.58		***
Anxious, not more than before Corona	8.07		12.27		7.29		***
Anxious, more than before Corona	21.68		24.64		21.12		***
Afraid of infection (foregone medical treatment)	12.17		13.15		11.99		*
*Coping appraisal: hope*							
Uplifting experience during Corona	71.04		61.45		72.82		***
Looking forward to sth after Corona	82.79		71.93		84.81		***
*Controls*							
Female	56.63		65.32		55.01		***
Migrant (foreign-born)	8.53		8.65		8.51		
Marital status							
Married/registered partnership	64.82		58.00		66.08		***
Never married	6.96		4.64		7.39		***
Divorced	10.41		6.12		11.20		***
Widowed	17.82		31.24		15.33		***
Household size							
Single	27.42		33.20		26.34		***
2 people	50.12		44.46		51.17		***
> 2 people	22.46		22.33		22.49		
Living in urban area	38.69		34.21		39.52		***
Home ownership	81.31		82.00		81.18		
Employment status							
Retired	53.70		67.00		51.23		***
(Self)employed	31.27		11.17		35.01		***
Unemployed	2.84		2.20		2.97		*
Sick/disabled	3.33		4.14		3.18		*
Homemaker	7.20		13.00		6.13		***
Other	1.64		2.49		1.49		***
Financial difficulties							
No financial difficulties	60.16		47.54		62.50		***
Experienced financial diff.	25.49		39.77		22.84		***
Severe financial difficulties	14.35		12.69		14.66		**
Education							
Primary education	31.58		54.04		27.41		***
Secondary education	39.17		29.75		40.92		***
Post-secondary education	29.25		16.21		31.67		***
Personality trait							
Openness	3.32 (0.93)		3.16 (0.89)		3.35 (0.93)		***
Conscientiousness	4.11 (0.79)		4.05 (0.82)		4.12 (0.79)		***
Extraversion	3.48 (0.92)		3.40 (0.89)		3.50 (0.92)		***
Agreeableness	3.67 (0.81)		3.63 (0.81)		3.67 (0.81)		**
Neuroticism	2.67 (1.00)		2.80 (0.97)		2.64 (1.01)		***
Trust in other people	5.91 (2.41)		5.63 (2.47)		5.96 (2.40)		***
Limited in leaving house	6.53		21.23		3.81		***
Controls from before Wave 8	26.66		29.42		26.14		***
**N**	**40,282**		**6,976**		**33,306**		

Data: SHARE Wave 8 Release 1.0.0 and Release 7.1.0. Oxford COVID-19 Government Response Tracker. Weighted data. * *p* < 0.05, ** *p* < 0.01, *** *p* < 0.001.

**Table 2.** Descriptive Statistics of National Conditions by Country.

Country	Stayed home: overall sample (N)	Prot Beh Index: left-home subsample (N)	Mortality: deaths per 100k (mean)	Oxford Stringency Index (mean)	Trust in health care system (mean)
Sweden (SE)	1150	1109	52.19	40.22	0.86
Denmark (DK)	1836	1800	10.36	58.91	0.87
Finland (FI)	895	857	5.94	40.81	0.94
Germany (DE)	2564	2465	10.81	51.24	0.90
Switzerland (CH)	1669	1483	22.74	54.29	n.a.
France (FR)	1737	1661	44.32	55.91	0.88
Belgium (BE)	3349	2987	84.22	53.44	0.97
Luxemburg (LU)	692	584	17.69	49.78	0.90
Italy (IT)	3116	2020	57.65	61.44	0.56
Spain (ES)	1581	1240	59.23	56.58	0.77
Portugal (PT)	851	698	15.77	58.45	0.55
Greece (GR)	2289	1679	1.82	60.54	0.26
Cyprus (CY)	288	178	2.13	69.24	0.73
Malta (MT)	623	333	1.75	50.52	0.94
Poland (PL)	1908	1712	3.90	49.35	0.32
Czech Republic (CZ)	2218	1983	3.27	44.26	0.78
Slovakia (SK)	759	664	0.51	46.98	0.50
Slovenia (SI)	2640	2198	5.29	54.71	0.73
Croatia (HR)	1631	1048	2.86	48.43	0.59
Hungary (HU)	713	516	5.98	59.53	0.47
Romania (RO)	1062	803	8.04	52.13	0.25
Bulgaria (BG)	647	498	4.35	44.53	0.29
Estonia (EE)	3419	2670	5.19	49.24	0.73
Latvia (LV)	629	537	1.60	44.19	0.47
Lithuania (LT)	1061	846	2.18	54.21	0.65
Israel (IL)	955	737	3.85	62.08	n.a.
**Overall N/Mean**	**40,282**	**33,306**	**16.68**	**52.73**	**0.66**

*Note.* Mean per country is calculated as average across June and July 2020 for mortality (fieldwork period) and since the outbreak of Corona until the end of July 2020 for stringency. Mean per country for trust in healthcare systems is based on Eurobarometer data from 2013. Countries are sorted along a north–west to south–east diagonal through Europe to make geographical closeness visible.

Data: SHARE Wave 8 Release 1.0.0. Oxford COVID-19 Government Response Tracker. Special Eurobarometer 411, Wave 80.2.

With regard to our indicators for threat and coping appraisal in the overall sample, the majority of respondents (83%) was not directly exposed to COVID-19 (measured as being or knowing someone who developed symptoms, was tested positively, was hospitalized, or knowing someone who died). Concerning the average change in confirmed case numbers (185 cases), we can bear in mind that they fluctuated between 0 and 8,618 during the time of fieldwork. In terms of vulnerability, 4% have three or more health risk conditions, and on average, 16% belong to the oldest age group. The fear arousal indicators show that 70% did not report any feelings of anxiousness. However, more than one in five respondents (22%) reported to feel more anxious than before COVID-19. On average, 12% are afraid of infection. Despite the circumstances, many older respondents expressed their hope through optimistic statements. About 71% named an uplifting and hope-inspiring experience during COVID-19, and over 80% look forward to something once COVID-19 abates.

In terms of demographic characteristics, the overall sample consists of 57% female and 9% foreign-born respondents, 27% live alone and 18% are widowed, 54% are retired, almost 30% achieved higher education levels, and 25% experienced severe financial difficulties due to the COVID-19 situation.

Table 1 also provides a description on the subsample that stayed home completely. We see that 36% were 80 years and older, and 21% of those who stayed home completely were limited in their mobility to leave the house independently already before COVID-19. Further cross-tabulations showed that the majority of the oldest age group left their home (64%), 22% stayed home with the onset of COVID-19 and not before, and 14% stayed home and were limited in leaving the house already before the outbreak of COVID-19.

### Protective Behavior

**Figure 2.**
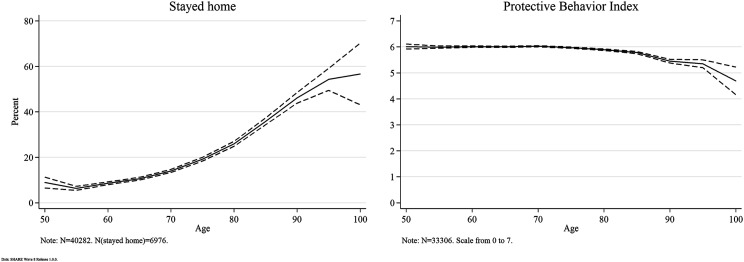
Protective behavior across age. Data: SHARE Wave 8 Release 1.0.0. Weighted data. 95% confidence interval.

Figure 2 shows how protective behavior varies across the age span from age 50 to 100. While the proportion of those staying home increases sharply with age from 6% among 55-year-olds to 54% among 95-year-olds, the amount of protective behavior measures is very stable until age 80 and then decreases only slightly.

**Figure 3.**
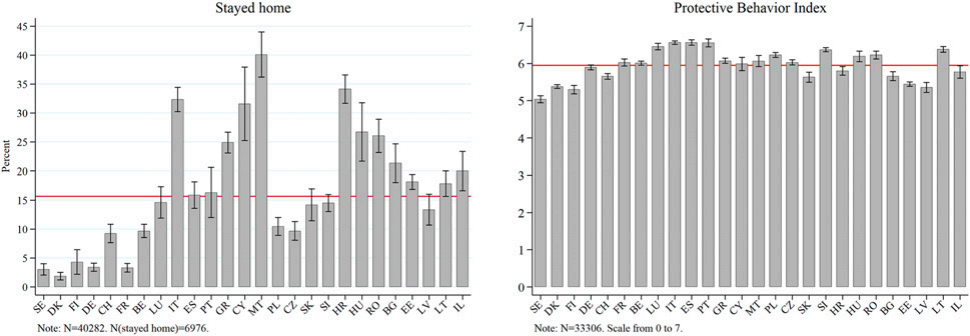
Protective behavior across Europe. Data: SHARE Wave 8 Release 1.0.0. Weighted data. 95% confidence interval. Controlled for age.

Even though the overall mean of those who stayed home since the outbreak of COVID-19 is around 15%, Figure 3 shows that the rates vary strongly by country. While 30 to 40% of all interviewed persons from Croatia, Cyprus, Italy, and Malta stayed home completely during the time of observation, this applies only to less than 5% in Denmark, Finland, Sweden, France, and Germany. Countries with low stay-home rates also show high degrees of trust in the existing national healthcare system (ranging from 86 to 94%). In contrast to that, Italy faced an especially uncertain situation with being the first severely hit country in Europe. Cyprus required all its citizens to send a text message to a governmental number to be able to leave the house (Cyprus Government, 2020). Malta introduced age-specific lockdown regulations. For instance, persons over 65 were not allowed to leave their homes unless absolutely necessary (Grech, 2020). In Croatia, a ban on leaving one’s place of residence was imposed during the lockdown in spring 2020 (Forjan, 2020).

Table 2 illustrates country-specific sample sizes and corresponding macrolevel indicators on COVID-19 mortality (Hale et al., 2021; Ritchie et al., 2020b), governmental stringency (Hale et al., 2021; Ritchie et al., 2020a), and trust in the healthcare system based on Eurobarometer data from 2013 (European Commission, 2014). It can be seen that some of the countries with a large share of people who stayed home implemented strict governmental control measures (Oxford stringency index > 50) such as Cyprus, Hungary, or Italy. However, the stringency index does not capture all regulations in detail.

Based on the subsample of those who left home, we see that all countries have very high average scores on the summary index of protective behavior. These rates deviate only slightly from the overall mean of 5.95, with the highest average scores in the southern countries, Italy, Portugal, and Spain (between 6.53 and 6.54). While Italy and Spain had comparably high death rates after the onset of the crisis, Portugal had one of the highest stringency levels. The lowest protective behavior means (between 5.13 and 5.47) can be seen in the northern and Baltic states (Denmark, Finland, Sweden, Estonia, and Latvia). With the exception of Latvia, in those countries, mask-wearing was not mandatory at the time of fieldwork (ECDC, 2021)^2^ and citizens display considerably high levels of trust in the national healthcare system (see Table 2).

**Figure 4.**
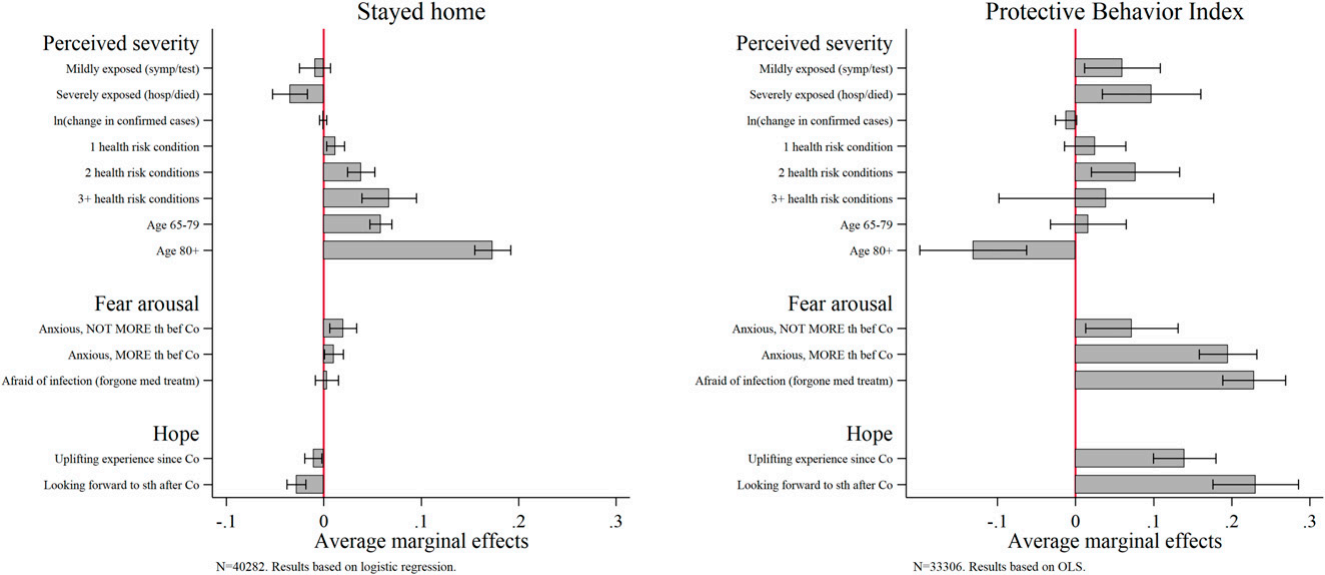
AMEs by PMT component for “Stayed home” and “Protective Behavior Index”. Data: SHARE Wave 8 Release 1.0.0 and Release 7.1.0. Oxford COVID-19 Government Response Tracker. Controlled for country, sex, migrant, marital status, household size, urban, home ownership, education, employment status, personality traits, trust, limited in leaving house, and controls from before Wave 8. Weighted data. 95% confidence interval. Age and health risk conditions enter the model as interaction.

To further answer our research questions, we perform multivariate logistic and linear regressions on our dependent variables “Stayed home” and “Protective Behavior Index.” Figure 4 displays all indicators for the PMT components threat appraisal and coping appraisal. We plot average marginal effects (AMEs) to be able to compare the size of the coefficients across all indicators (Mood, 2010).

### Who Stays Home?

The left panel of Figure 4 shows that perceived vulnerability is the main driving factor to stay at home. More precisely, this applies to persons above age 65, especially the oldest old (AME = .173, *p* < .001), and is higher for respondents with three or more health risk conditions (AME = .067, *p* < .001). Apart from those who feel vulnerable, respondents who report feelings of anxiousness (that are not COVID-19–specific) tend to stay home as well (AME = .020, *p* < .01). In contrast to that, interviewed persons who report severe exposure to the virus (AME = −.034, *p* < .001) and who express hope (AME = −.011, *p* < .01 for uplifting experience; AME = −.028, *p* < .001 for looking forward) are rather those who left their home.

With respect to sociodemographic control variables, we find that being female, not working due to sickness or disability, lower educational levels, experiencing financial difficulties, and limitations in leaving the house independently before COVID-19 are strong and significant predictors for staying home (*p* < .001). However, belonging to the age group 80+ shows by far the strongest association for all other covariates (see Table A1 in the Online Appendix). We controlled for limitations in leaving the house, which only slightly reduced the AME for the age group 80+. But we cannot further distinguish if the oldest old stayed home due to COVID-19 or due to alternative causes (i.e., selection) as we observe associations only. Since McFadden’s Pseudo-R^2^ is at 0.217 for the logistic regression on “Stayed home,” we consider our model fit as very good (according to McFadden, 1978).

### What Motivates Protective Behavior?

The right panel of Figure 4 shows the results for the subsample of respondents who left their home. Apparently, fear arousal due to COVID-19 and hope are the main motivators for adopting protective behavior. Three of the five indicators (“Anxious, more than before Corona,” “Afraid of infection,” and “Looking forward to something after Corona”) show equally strong associations of around .2 and are all significant (*p* < .001). In contrast, the AMEs are smaller for respondents who are actually exposed to the virus (mildly: AME = .060, *p* < .05; severely: AME = .097, *p* < .01). The AME for potential exposure indicated by the change in confirmed cases is insignificant. The only significant negative association can be seen among the oldest old who appear to be less motivated or able to apply the recommended protective measures (AME = −.131, *p* < .001).

Other decisive determinants of protective behavior are shown in Table A1 in the Online Appendix. They are being female (AME = .184, *p* < .001), born in a foreign country (AME = .158, *p* < .001), living in a two-person household (AME = .118, *p* < .001), reporting financial difficulties (AME = .086, *p* < .001), and having attained higher education levels (AME = .138, *p* < .001). In terms of personality traits, persons with higher degrees of conscientiousness (e.g., being disciplined and careful; AME = .069, *p* < .001) and neuroticism (e.g., being anxious and pessimistic; AME = .042, *p* < .001) take up more protective behaviors. In total, 16.4% of the total variance of the regression on the “Protective Behavior Index” can be explained by the covariates included in the model as stated by the R^2^ value in Table A1.

### Perceived Severity and Perceived Vulnerability

**Figure 5.**
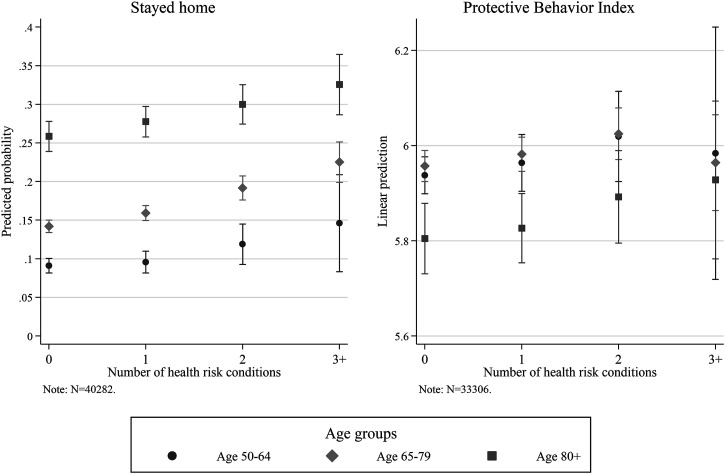
Vulnerability interaction for “Stayed home” and “Protective Behavior Index.” *Note.* Based on logistic regression on Stayed home and OLS on Protective Behavior Index. 95% confidence interval. Weighted data. Data: SHARE Wave 8 Release 1.0.0 and Release 7.1.0. Oxford COVID-19 Government Response Tracker.

As indicated above, our perceived severity indicator that captures the change in confirmed cases does not play a significant role, whereas our measure for self- or network-exposure to the virus is significantly associated with more self-protective behaviors. In our model, perceived vulnerability is a combination of health risk conditions and age. Based on the interaction term, Figure 5 shows the predicted probability to stay home (left panel) and the linear predictions to take up a specific amount of protective measures (right panel) for individuals from different age groups across the number of health risk conditions. Overall, we can see that the oldest old are most likely to stay home and that the likelihood increases with the number of health risk conditions for all age groups. Against our expectations, none of the interaction terms stands out significantly. The most vulnerable people aged over 80 with three or more risk conditions, have a predicted probability to stay home of 33%, in contrast to 9% for a healthy, professionally active person below age 64 with none or one risk condition. The right panel of Figure 5 reveals that the age groups below 80 show similar rates—independent of their health risk conditions status. Here, the healthy oldest old show the lowest rates, which could well be a selection of the subsample of those who left their home.

### Fear Arousal versus Hope

The main motivating factors for adopting protective behavior in the “Protective Behavior Index left-home subsample” model are fear and hope. In the following analyses, we investigate the country-specific influence of fear and hope by introducing additional interaction terms with country dummies for all variables in the model (fully interacted model). We expect associations to vary depending on country-specific death rates, containment strategies, welfare and healthcare systems, and unobserved variation in governmental or media communication and due to cultural aspects.

**Figure 6.**
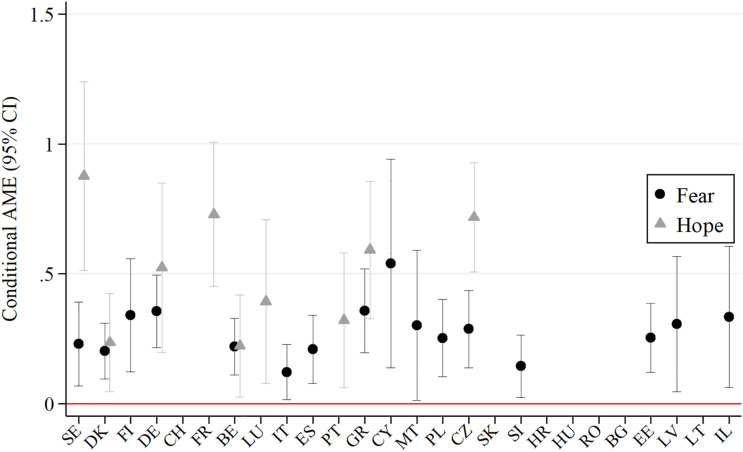
Significant AMEs for fear and hope by country. *Note.* Based on the regression model on Protective Behavior Index, fully interacted with country. Significant estimates only (95% level). Countries in geographical order. *N* = 33,306. Data: SHARE Wave 8 Release 1.0.0 and Release 7.1.0. Oxford COVID-19 Government Response Tracker.

**Figure 7.**
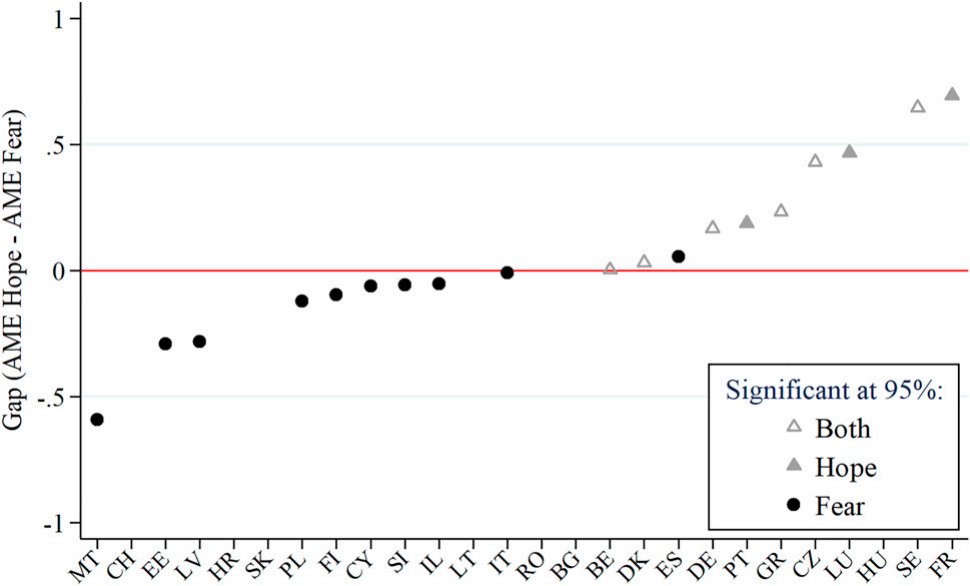
Fear–hope gap by country. *Note.* Based on the regression model on Protective Behavior Index, fully interacted with country. Countries in ascending order. *N* = 33,306. Data: SHARE Wave 8 Release 1.0.0 and Release 7.1.0. Oxford COVID-19 Government Response Tracker.

Figure 6 shows the conditional AMEs of “Anxious, more than before Corona” by country (black dots, labeled “fear”) and the variable “Looking forward to sth after Corona” by country (gray triangles, labeled “hope”). For the sake of clarity, only significant interaction terms are displayed (*p* < .05). Overall, 16 out of 26 countries show a positive significant association of feeling more anxious than before COVID-19 with protective behavior, and 9 out of 26 countries show a positive significant association with hope. With .3 or more, the largest AMEs for fear can be observed in Cyprus, Greece, Germany, and Finland. In contrast, the largest AMEs for hope are shown in Sweden, France, the Czech Republic, and Greece. In Figure 7, all countries are sorted by the difference between the AMEs for fear and hope in an ascending order. Hence, the figure reflects the fear–hope gap. Based on significance and magnitude of the AME, we can distinguish three patterns: (1) countries with a dominant fear pattern (especially Malta, Estonia, and Latvia, and less strong Poland, Finland, Cyprus, Slovenia, Israel, Italy, and Spain), (2) countries with an equally strong influence of both indicators (Belgium, Denmark, Germany, and Greece), and (3) countries with hope as the dominant pattern (France, Sweden, Luxembourg, and the Czech Republic, and less strong, Portugal). By drawing on the national conditions in Table 2, it can be seen that with the exception of Italy and Spain, all countries with a dominant fear pattern are among the countries with low COVID-19 mortality rates (< 6 deaths per 100,000 inhabitants) and that with the exception of Portugal, the countries with a dominant hope pattern show high or very high trust in their healthcare system (78–90%).

### Sensitivity Analyses and Robustness Checks

As we use an additive index for our continuous dependent variable “Protective Behavior Index,” we examined several alternative outcome specifications and modeling strategies to check the robustness of our findings and extend the main findings where needed. Our sensitivity analyses and robustness checks provide very similar estimates with regard to significance and coefficient size. The results are especially stable for our main indicators of interest—fear and hope. Substantively, the additive index combines behaviors from different domains. While it only contains behaviors that were officially recommended in all countries (besides mask-wearing in Finland), they can be subdivided into avoidant and preventive behavior on a theoretical ground (as outlined in the section on measures). Factor analysis yields three factors, which can be labeled as contact reduction, distance and mask, and hygiene measures. Regressions on these three factor scores point to important differences among the age group 80+, but conceal important differences in other variables of interest. Table A2 in the Online Appendix takes these disparities into account by showing the regression results for each item of the protective behavior index separately. We added the aforementioned theoretical and factor analytical distinctions in the upper two rows of the table to enable an adequate interpretation of the results.

Compared to the results mentioned above, we see the following differences regarding our PMT indicators. In terms of perceived severity, we observed a significant positive association of severe exposure to the virus and the Protective Behavior Index. The breakdown by items shows that this result is driven by physical distancing and using sanitizer and is unrelated to theoretical or factor-analytical sorting. Regarding perceived vulnerability, the most vulnerable age group (80+) is not only most likely to stay at home completely, but also shows a higher likelihood to reduce any physical contact (in terms of visiting family and meeting with others). In contrast, this age group is significantly less likely to adopt any of the hygiene measures or adhere to physical distancing in general. With regard to fear arousal, the coefficients for feeling anxious due to COVID-19 remain stable across all specifications. The same holds for hope.

Further sensitivity analyses refer to our modeling strategy. The Protective Behavior Index consists of seven behaviors, which result in an additive index ranging from 0 to 7 (count variable). As most people comply with the recommended measures (mean, 5.95; median, 6), the distribution is left-skewed (skewness, −1.40; kurtosis, 5.25) and not normally distributed. Log transformation did not improve the distribution (skewness, −2.68; kurtosis, 14.15). An alternative modeling strategy for highly right-skewed count distributions (with many zeros) are Poisson models or negative binomial regression models (NBRM). In order to illustrate the robustness of our findings based on linear ordinary least squares (OLS) regression, Table A3 in the Online Appendix shows our chosen OLS model in comparison with (a) a reversed Protective Behavior Index to mirror the right-skewed distribution of count models, and (b) the results of a negative binomial regression model on the reversed Protective Behavior Index. The results demonstrate that all types of models provide very similar estimates with regard to significance and coefficient size. Therefore, we report our results based on the basic linear OLS estimation using an additive index.

## Discussion and Conclusion

This study explored the relationship of fear and hope with older people’s intention to engage in self-protective behavior during the initial phase of the COVID-19 crisis, controlling for sociodemographic background, intrapersonal characteristics, and contextual variables. Toward this end, we used data from the SHARE Corona Survey and drew on the psychometric paradigm and protection motivation theory as our theoretical foundation. Our major findings are as follows.

In the period under review, SHARE respondents’ potential exposure to the virus was considerably low in most countries, as indicated by the change in the number of confirmed cases from the previous to the interview day (on average 185) and the death rate per 100,000 inhabitants (on average 17). In spite of that, respondents’ engagement in the recommended self-protective behavior was remarkably high and in compliance with the governmental containment and mitigation strategies. We explain this by drawing on protection motivation theory’s components threat and coping appraisal and the corresponding indicators. We found that around 15% of older Europeans stayed home completely in the first months after the outbreak of the virus. In line with H2 (vulnerability hypothesis), the vulnerable group of oldest old, those with prior health risk conditions, and partly those living in countries with comparably strict governmental control measures (policy stringency index above average in countries such as Cyprus, Hungary, or Italy) stayed home most. In this regard, the results for Malta (potentially also Italy, Cyprus, and Croatia) should be treated with caution because they implemented the policy that specific age groups were not allowed to leave home.

If respondents did not stay at home for any of the reasons mentioned above, we observed a significantly higher uptake of protective behaviors among those respondents who were actually affected by the virus (i.e., personally mildly or severely exposed or knowing someone exposed). Since respondents’ behavior was associated with actual exposure but not with the change in the number of confirmed cases, we can only partly confirm H1 (severity hypothesis). This is in line with the concept of network-exposure severity by Litwin and Levinsky (2021). The authors explain that people’s behavior is influenced primarily by feedback from trusted members of their social networks. Therefore, knowing someone affected by the virus may motivate self-protective behavior more than receiving information about the virus from unknown others or the public.

Our most striking finding is that fear and hope as our PMT measures for threat and coping appraisal function as opposing motivational factors. On the one hand, feeling more anxious than before COVID-19 and fear of infection were strong motivators for protective behavior, confirming H3 (fear hypothesis). Interestingly, we observed this in many countries with low COVID-19 mortality rates such as Malta, Estonia, Latvia, Poland, or Finland (see Table 2). On the other hand, hope shows an alternative pathway to fear and corroborates H4 (hope hypothesis). The association for respondents who report any uplifting experience since the outbreak of COVID-19 or mention anything they look forward to after the crisis with protective behavior is equally strong as with fear. Countries where fear did not have a significant influence and where we found the largest predictions for hope are France and Luxembourg. Hope outweighs fear in Sweden and the Czech Republic. In accordance with prior considerations on the role of “greater trust in the societal mechanisms that handled the pandemic” by Litwin and Levinsky (2021, p. 19), we showed that most of these countries have above-average levels of trust in their healthcare systems (see Table 2). Another contradictory result was seen for the population 80+. While the oldest old were most likely to stay home completely, those who left home engaged significantly less in keeping distance, wearing a face mask, and hygienic measures. They might have more experience with crisis situations and therefore have a different threat perception. In line with Charles (2010), their coping strategies could be more flexible due to the experience, knowledge, and positivity gained throughout life. Alternatively, they might not be fully capable of applying or adapting the constantly changing regulations. Another explanation is that they are more fatalistic with regard to their quality of life and end of life (Litwin & Levinsky, 2021).

Some limitations of our study should be acknowledged. First, we treat COVID-19 as a health threat, but cannot distinguish the underlying motivation for protective behavior. While we address fear of infection, we cannot distinguish further potential underlying motivators such as fear of spreading the virus and infecting others, fear of public shaming, or fear of conflict with public officials due to non-compliance with governmental rules. Second, our Protective Behavior Index does not capture how well respondents adhered to recommended behaviors in relation to a national benchmark. While we deliberately rely on recommended protective behaviors only, we are aware that taking into account the variation in regulations across countries and their change over time would be more precise (e.g., stay home obligations). Third, we cannot distinguish further between the reasons of staying home—if respondents stayed home in order to follow the stay-at-home-orders, to protect themselves from the virus, due to lack of alternative places to go in times of lockdown, or due to health conditions that made them stay at home already prior to COVID-19. We tried to capture some of this noise by using a control variable on restricted mobility (i.e., limitations in leaving the house independently prior to COVID-19), but cannot account for reasons any further. Fourth, our indicator number of health risk conditions for perceived vulnerability was measured prior to the outbreak of the virus and may have changed since then. Another limitation is based on the suggestive question wording of both hope indicators in the SHARE Corona Survey. Respondents were asked to name an uplifting and hope-inspiring experience and something to look forward to. Even though they had the option to remain silent, respondents might have felt prompted to name something, regardless of whether they were hopeful or not. Finally, we cannot draw causal conclusions. So far, we can only make statements about associations and cannot rule out reverse causality in the relationship between perceived severity, perceived vulnerability, fear arousal, and hope with protective behavior. According to PMT, our control variables are anticipatory covariates and measured prior to the outbreak of COVID-19, whereas all PMT components and the outcome variable protective behavior are in a unidirectional relationship and measured at the same point in time after the outbreak of COVID-19. We hope that the follow-up SHARE Corona Survey can shed light on the direction of the relationships.

Further research is needed in conceptualizing and analyzing the manifold threat dimensions of COVID-19. While the data at hand allowed us to focus on COVID-19 as a concrete health threat, future studies should consider alternative threat scenarios directed toward economic hardship, stability of political and economic systems, and changing dynamics in socio-psychological behaviors (see study by Kachanoff et al., 2020) on realistic and symbolic threats of COVID-19).

In line with extant research (e.g., Graef & Henning, 2020; Liu & Liu, 2020), further research should account for media attention, media coverage, and governmental communication strategies because of their influence on people’s threat perceptions and coping appraisal, for instance, through drastic language and war metaphors. While fear strongly motivates people in the short run, research documented manifold negative health consequences of fear in the long run (e.g., Nechita et al., 2018; Seiler et al., 2020). In contrast, this study pointed out that hope can even trump fear. Governmental communication predominantly reaches citizens through press and media, especially older people as the most affected social group with the highest (traditional) media and news consumption (Papathanassopoulos et al., 2013). According to media and communication research, messages of hope in news stories can be as effective as messages of fear in promoting protective behavior (Nabi & Prestin, 2016). Therefore, decision-makers should consider a healthy balance between instrumenting fear as motivational factor and hope as healthy long-term motivator in times of crisis. With regard to NPIs and targeted communication, Balinska and Rizzo (2009) stress that governments’ decisions have to be well-balanced because social disruptions due to conflicting recommendations, insufficient medical staff, media-induced panic, and media censorship tend to increase with the length of imposed protection measures. They conclude that an “adequate and transparent information on the part of health care authorities and in collaboration with the media, business, and organizations will be paramount for disease control and containment” (p. 9). An adequate communication approach could address the sovereignty of citizens; envision optimistic post-crisis scenarios; and ensure an honest, informative, and transparent communication. Given the negative health effects of fear, our findings may help evaluate and revise governmental policy responses and communication strategies in an enduring crisis.

## Supplemental Material

Supplemental Material - Motivating Protective Behavior against COVID-19: Fear Versus Hope Supplemental Material for Motivating Protective Behavior against COVID-19: Fear Versus Hope by Gregor Sand, and Johanna Bristle in Journal of Aging and Health

## Notes

1. According to the WHO, a confirmed case refers to a “person with laboratory confirmation of infection with the novel coronavirus” (WHO, 2020).

2. In Estonia and Denmark, mask-wearing was recommended throughout the whole fieldwork period. In Sweden, masks were recommended since 1 July 2020 (half-time of fieldwork). Finland is the only country where masks were not recommended nor mandatory during fieldwork. The only other country without mandatory mask regulations was Switzerland (masks were recommended). In all other SHARE countries, mask-wearing was mandatory during the entire time of fieldwork (ECDC, 2021).
