# Supplementary material for: Motivating Protective Behavior against COVID-19: Fear Versus Hope
Source: J Aging Health. 2022 Jun 17;36(5-6):350–66. doi: 10.1177/08982643221089427 (PMC9207583; doi:10.1177/08982643221089427)
Supplement: Supplemental Material - Motivating Protective Behavior against COVID-19: Fear Versus Hope [file sj-pdf-1-jah-10.1177_08982643221089427.pdf]

Online Appendix –
Supplement to "Motivating Protective Behavior against COVID-19: Fear vs. Hope"

Table A1. Full Regression Models on Stayed home and Protective Behavior Index (Average Marginal Effects)

	Stayed home		Protective Beh Index	
Threat appraisal: perceived severity				
Exposure (ref: not exposed)				
Mildly exposed (symptoms/tested positive)	-0.009	(0.01)	0.060*	(0.03)
Severely exposed (hospitalized/died)	-0.034***	(0.01)	0.097**	(0.03)
ln(change in confirmed cases)	-0.000	(0.00)	-0.011	(0.01)
Threat appraisal: perceived vulnerability				
Number of health risk conditions (ref: no)				
1 health risk condition	0.012**	(0.00)	0.025	(0.02)
2 health risk conditions	0.038**	(0.01)	0.077**	(0.03)
3+ health risk conditions	0.067**	(0.01)	0.039	(0.07)
Age (ref: 50-64)				
65-79	0.059***	(0.01)	0.016	(0.02)
80 +	0.173***	(0.01)	-0.131***	(0.03)
Threat appraisal: fear arousal				
Feeling anxious (ref: not anxious)				
Anxious, not more than before Corona	0.020**	(0.01)	0.072*	(0.03)
Anxious, more than before Corona	0.010*	(0.00)	0.195***	(0.02)
Afraid of infection (foregone medical treatment)	0.003	(0.01)	0.229***	(0.02)
Coping appraisal: hope				
Uplifting experience during Corona	-0.011*	(0.00)	0.140***	(0.02)
Looking forward to sth after Corona	-0.028***	(0.01)	0.231***	(0.03)
Controls				
Female	0.027***	(0.00)	0.184***	(0.02)
Migrant (foreign-born)	0.002	(0.01)	0.158***	(0.03)
Marital status (ref: married/registered partnership)				
Never married	-0.017	(0.01)	-0.211***	(0.05)
Divorced	-0.021*	(0.01)	-0.149***	(0.04)
Widowed	0.003	(0.01)	-0.100**	(0.03)
Household size (ref: single)				
2 people	-0.001	(0.01)	0.118***	(0.03)
>2 people	0.017*	(0.01)	0.062	(0.03)
Living in urban area	-0.019***	(0.00)	0.040*	(0.02)
Home ownership	-0.011*	(0.01)	0.071**	(0.02)
Employment status (ref: retired)				
(Self)employed	-0.039***	(0.01)	-0.015	(0.03)
Unemployed	0.005	(0.02)	-0.144*	(0.06)
Sick/disabled	0.050**	(0.02)	-0.041	(0.06)
Homemaker	0.013	(0.01)	0.003	(0.03)
Other	0.015	(0.02)	-0.099	(0.07)
Financial difficulties (ref: none)				
Experienced financial difficulties	0.018***	(0.00)	0.086***	(0.02)
Severe financial difficulties	0.008	(0.01)	0.059*	(0.03)
Education (ref: primary)				
Secondary	-0.035***	(0.01)	0.097***	(0.02)
Post-secondary	-0.041***	(0.01)	0.138***	(0.02)
Personality trait – Openness	-0.005*	(0.00)	0.001	(0.01)
Personality trait – Conscientiousness	-0.004**	(0.00)	0.069***	(0.01)
Personality trait – Extraversion	-0.002	(0.00)	0.003	(0.01)
Personality trait – Agreeableness	0.001	(0.00)	0.007	(0.01)
Personality trait – Neuroticism	-0.001	(0.00)	0.042***	(0.01)
Trust in other people	0.000	(0.00)	0.012**	(0.00)
Limited in leaving the house before Corona	0.114***	(0.01)	-0.156***	(0.04)
Controls from before Wave 8	0.019***	(0.01)	0.016	(0.02)
N	40,282		33,306	
Pseudo-R^2 / R^2	0.217		0.164	

Note: Weighted sample. Average marginal effects reported. Robust standard errors in parentheses. Controlled for country. Age and health risk conditions enter the model as interaction. * $p < 0.05$, ** $p < 0.01$, *** $p < 0.001$.
Data: SHARE Wave 8 Release 1.0.0 and Release 7.1.0. Oxford COVID-19 Government Response Tracker.

Table A2. Item-Specific Regressions with Thematic Clustering. Logistic Regression on each Protective Behavior Separately (Average Marginal Effects)

Theoretical distinction	Avoidant behavior			Preventive behavior			
Types based on factor analysis	Contact reduction		Distance and mask		Hygiene measures		
Outcome	Meet 5+ people less/not	Visit family less/not	Keep distance	Wear mask	Wash hands	Use sanitizer	Cover cough/ sneeze
Threat appraisal: Perceived severity							
Exposure (ref: not exp)							
Mildly exposed	0.014	0.015	0.008	0.011	-0.004	-0.002	0.015
(symptoms/tested pos)	(0.01)	(0.01)	(0.01)	(0.01)	(0.01)	(0.01)	(0.01)
Severely exposed	0.024*	-0.001	0.018**	0.019*	0.006	0.034***	0.004
(hospitalized/died)	(0.01)	(0.01)	(0.01)	(0.01)	(0.01)	(0.01)	(0.01)
ln(change in confirmed	-0.003	-0.002	-0.002	0.005*	-0.002	-0.004*	-0.005*
cases)	(0.00)	(0.00)	(0.00)	(0.00)	(0.00)	(0.00)	(0.00)
Threat appraisal: Perceived vulnerability							
Number of health risk							
conditions (ref: no)							-
1 health risk cond.	0.001	0.018**	0.002	0.009	0.003	0.005	0.006
	(0.01)	(0.01)	(0.00)	(0.01)	(0.01)	(0.01)	(0.01)
2 health risk cond.	0.003	0.026**	0.005	0.015	0.007	0.009	0.010
	(0.01)	(0.01)	(0.00)	(0.01)	(0.01)	(0.01)	(0.01)
3+ health risk cond.	-0.002	0.043*	-0.035	0.007	0.000	0.011	0.029
	(0.02)	(0.02)	(0.02)	(0.02)	(0.02)	(0.01)	(0.02)
Age (ref: 50-64)							
Age 65-79	0.035***	0.038***	-0.003	0.005	0.001	-0.026***	-0.026***
	(0.01)	(0.01)	(0.00)	(0.01)	(0.01)	(0.01)	(0.01)
Age 80+	0.046***	0.053***	-0.025***	0.000	-0.013	-0.075***	-0.080***
	(0.01)	(0.01)	(0.01)	(0.01)	(0.01)	(0.01)	(0.01)
Threat appraisal: Fear arousal							
Feeling anx (ref: not)							
Anxious, not more	0.006	0.012	0.005	-0.005	0.009	0.016*	0.026***
than before Corona	(0.01)	(0.01)	(0.01)	(0.01)	(0.01)	(0.01)	(0.01)
Anxious, more than	0.035***	0.052***	0.016***	0.021***	0.027***	0.026***	0.025***
before Corona	(0.01)	(0.01)	(0.00)	(0.01)	(0.01)	(0.01)	(0.01)
Afraid of infection	0.048***	0.049***	0.037***	0.036***	0.032***	0.028***	0.029***
(foregone med treatm)	(0.01)	(0.01)	(0.01)	(0.01)	(0.01)	(0.01)	(0.01)
Coping appraisal: Hope							
Uplifting experience	0.010	0.006	0.010**	0.015**	0.034***	0.025***	0.028***
during Corona	(0.01)	(0.01)	(0.00)	(0.01)	(0.00)	(0.01)	(0.01)
Looking forward to sth	0.034***	0.047***	0.017***	0.013	0.022***	0.031***	0.016*
after Corona	(0.01)	(0.01)	(0.00)	(0.01)	(0.01)	(0.01)	(0.01)
N	32,600	32,612	33,240	31,914	33,265	33,300	33,140

Note: Weighted sample. Average marginal effects reported. Robust standard errors in parentheses. * $p < 0.05$, ** $p < 0.01$, *** $p < 0.001$. Controlled for country, sex, migrant, marital status, household size, urban, home ownership, employment status, financial difficulties, education, personality traits, trust, limited in leaving house, controls from before Wave 8. Age and health risk conditions enter the model as interaction.

Data: SHARE Wave 8 Release 1.0.0 and Release 7.1.0. Oxford COVID-19 Government Response Tracker.

Table A3. Specifications and Modelling Strategies for Protective Behavior Index (PBI): Ordinary Least Squares (OLS) vs. Negative Binomial Regression Model (NBRM)

	OLS on PBI	(a) OLS on reversed PBI	(b) NBRM on reversed PBI
Threat appraisal: Perceived severity			
Exposure (ref: not exp)			
Mildly exposed (symptoms/tested positive)	0.060*	-0.060*	-0.051*
	(0.025)	(0.025)	(0.024)
Severely exposed (hospitalized/died)	0.097**	-0.097**	-0.108**
	(0.032)	(0.032)	(0.037)
ln(change in confirmed cases)	-0.013	0.013	0.013
	(0.007)	(0.007)	(0.007)
Threat appraisal: Perceived vulnerability			
Number of health risk conditions (ref: no)			
1 health risk condition	0.026	-0.026	-0.034
	(0.034)	(0.034)	(0.033)
2 health risk conditions	0.082	-0.082	-0.081
	(0.051)	(0.051)	(0.053)
3+ health risk conditions	0.046	-0.046	-0.043
	(0.137)	(0.137)	(0.119)
Age (ref: 50-64)			
Age 65-79	0.020	-0.020	0.011
	(0.029)	(0.029)	(0.028)
Age 80+	-0.133***	0.133***	0.135***
	(0.045)	(0.045)	(0.041)
Threat appraisal: Fear arousal			
Feeling anxious (ref: not)			
Anxious, not more than before Corona	0.072*	-0.072*	-0.062*
	(0.030)	(0.030)	(0.030)
Anxious, more than before Corona	0.195***	-0.195***	-0.210***
	(0.019)	(0.019)	(0.021)
Afraid of infection (foregone medical treatment)	0.229***	-0.229***	-0.245***
	(0.020)	(0.020)	(0.024)
Coping appraisal: Hope			
Uplifting experience during Corona	0.140***	-0.140***	-0.130***
	(0.020)	(0.020)	(0.018)
Looking forward to sth after Corona	0.231***	-0.231***	-0.199***
	(0.028)	(0.028)	(0.023)
N	33,306	33,306	33,306
R^2	0.164	0.164	

Note: Weighted sample. Beta coefficients reported. Robust standard errors in parentheses. * $p < 0.05$, ** $p < 0.01$, *** $p < 0.001$. Controlled for country, sex, migrant, marital status, household size, urban, home ownership, employment status, financial difficulties, education, personality traits, trust, limited in leaving house, controls from before Wave 8. Age and health risk conditions enter the model as interaction.

Data: SHARE Wave 8 Release 1.0.0 and Release 7.1.0. Oxford COVID-19 Government Response Tracker.
